# Pharmacokinetics and tolerability of the dual TORC1/2 inhibitor sapanisertib in combination with the MEK inhibitor trametinib in dogs

**DOI:** 10.3389/fvets.2022.1056408

**Published:** 2022-12-14

**Authors:** Bih-Rong Wei, Cody J. Peer, William J. Richardson, Stephen M. Hewitt, William D. Figg, R. Mark Simpson

**Affiliations:** ^1^Laboratory of Cancer Biology and Genetics, Center for Cancer Research, National Cancer Institute, Bethesda, MD, United States; ^2^Leidos Biomedical Research, Inc., Frederick National Laboratory for Cancer Research, Frederick, MD, United States; ^3^Clinical Pharmacology Program, Center for Cancer Research, National Cancer Institute, Bethesda, MD, United States; ^4^Laboratory of Pathology, Center for Cancer Research, National Cancer Institute, Bethesda, MD, United States; ^5^Genitourinary Malignancies Branch, Center for Cancer Research, National Cancer Institute, Bethesda, MD, United States

**Keywords:** kinase inhibition, combination therapy, comparative oncology, translational research, drug-drug interaction, melanoma, veterinary, drug safety

## Abstract

Activation of one or both the Ras/MAPK and PI3K/Akt/mTOR signal transduction pathways are known to mediate oncogenicity of several canine and human cancers, including mucosal melanomas. Reciprocal cross activation between the two pathways can be a source of drug resistance. Consequently, oral dosing for plasma pharmacokinetic (PK) analysis and tolerability to a combination of sapanisertib, a dual TORC1/2 inhibitor, and trametinib, a MEK inhibitor, was evaluated in nontumor-bearing laboratory dogs for its potential application in parallel pathway targeting. Twelve dogs, divided into three equal cohorts, received either the combination or single agents. Animals were monitored for PK following single dose and 17-day repeat dosing, and by clinical observations, hematology, serum biochemistry, coagulation studies and urinalyses. A single trametinib dose (0.025 mg/kg), sulfated as dimethyl sulfoxide which enhanced its absorption, reached mean maximum concentration (C_max_) 0.64 ng/mL [18% coefficient of variation (CV)] at a median time to maximum concentration (T_max_) of 1.5 h (hr), and mean area under the concentration-time curve (AUC) 16.8 hr^*^ng/mL (14%CV), which were similar when given alone or in combination with sapanisertib. A prolonged half-life afforded 3–4-fold plasma accumulation of trametinib with daily dosing, analogous to humans. Trametinib PK mirrored previous regulatory data in dogs, while exposure approximated some published human values but generally not all patients. Sapanisertib-alone in canine plasma following single 0.1 mg/kg dose [mean C_max_ 26.3 ng/mL (21%CV), median T_max_ 2.0 hr, and mean AUC 248 hr^*^ng/mL (41%CV)] resembled levels in human therapeutic trials; whereas canine sapanisertib exposure was reduced when combined with trametinib, a known cytochrome P450 CYP3A4 inducer. Sex differences were not observed for either drug. Side effects upon repeat dosing with either or both drugs may include body weight loss, maldigestion, and cutaneous discoloration. The combination was tolerated without dose limiting toxicity, although clinical laboratory analyses revealed drug-induced acute-phase inflammation, proteinuria, and decreased blood reticulocytes, mild changes not necessitating intervention. Short-term results in dogs with this combination would appear to hold translational promise for clinical trial evaluation to target canine and possibly human melanoma, as well as other cancers having one or both signal transduction pathway activations.

## Introduction

Targeted small molecule inhibitor drugs are increasingly used as alternatives and adjuncts to classical cytotoxic chemotherapeutics to treat dogs with spontaneous naturally occurring cancer, analogous to human cancer patients (1–3). By targeting cancers with greater specificity, patient response and safety profiles can be optimized. Additionally, some deleterious consequences of broader-spectrum chemotherapy may be avoided (4). A variety of small molecule drugs are approved for cancer care in humans and veterinary patients (e.g., canine therapeutics: toceranib phosphate and tigilanol tiglate), which have certain advantages as to stability, cost, patient compliance and pharmacokinetic (PK) properties (4, 5). Contemporary small molecule oncology drug targets include protein kinases, DNA damage repair enzymes, proteosomes and other proteins regulating epigenetic modifications. Such small molecule inhibitors are not without limits, however. Not all patients respond to these treatments and patients may acquire drug resistance after initial response (6). New treatment strategies must be developed based on the characteristics of the cancer cells and their tissue microenvironment, to guide therapy selection from among a variety of mechanisms of action to achieve the most optimal responses.

Oncogenic dysregulation of critical signaling pathways can lead to abnormal protein interaction cascades that alter cell growth, differentiation, metabolism, proliferation, survival, and motility. Unrestrained signaling events in cancer may be stimulated by growth factors, cytokines, cell-cell interactions, and cell-matrix interaction, as well as through gene mutational events (1). Two fundamental signal transduction pathways that may become dysregulated in a variety of cancers are RAS/MAPK (mitogen activated protein kinase) and phosphoinositide 3-kinase (PI3K)/Akt (protein kinase B)/mTOR (mammalian target of rapamycin). MAPK hyperactivity is a feature in up to 85% of human cancers (7), while the PI3K-Akt pathway is also frequently aberrantly activated in many human cancers (8). Enhanced cell signaling along these pathways have also been observed in canine cancers such as osteosarcoma, mast cell tumor, urothelial bladder cancer and melanoma (1, 9, 10). Overactivation of multiple mediators along one, or both, of these signaling pathways have been documented in canine mucosal melanoma, a feature that is shared with this rare melanoma subtype in people (11, 12). Furthermore, signaling cascade crosstalk occurs between the Ras/MAPK and PI3K/Akt/mTOR pathways and may serve as one mechanism of drug resistance in targeted monotherapy approaches (6, 13, 14). Therefore, combined targeting of RAS/MAPK and PI3K/Akt/mTOR may be beneficial for both canine and human mucosal melanomas (12, 13, 15). As canine spontaneous mucosal melanoma occurs relatively more frequently than does human mucosal melanoma, comparative oncology research conducted in canine patients has potential translational utility (11, 16).

Through a process of drug screening focusing on Ras/MAPK and PI3K/Akt/mTOR signal transduction pathways using canine mucosal melanoma cell lines and preclinical xenograft models, small molecule kinase inhibitors sapanisertib and trametinib were chosen for further evaluation as a combination (13). Sapanisertib (PubChem CID 45375953, or TAK-228), is an orally bioavailable benzoxazole inhibitor of raptor-mTOR (TORC1) and rictor-mTOR (TORC2), (mammalian target of rapamycin complex) within the PI3K/Akt signal transduction pathway acting through highly selective competitive adenosine triphosphate binding (17). Currently the subject of human oncology clinical trials for multiple cancer types, sapanisertib suppresses PI3K downstream mediators S6 and 4EBP1 in association with apoptosis and cell cycle arrest in canine melanoma cells (13). Trametinib (MEKINIST^®^), is a reversible non-receptor tyrosine kinase inhibitor targeting activated mitogen-activated protein/extracellular signal-regulated kinase 1 and 2 (MEK1/2). Trametinib is FDA-approved for the treatment of human patients with unresectable or metastatic melanoma harboring BRAF (v-raf murine sarcoma viral oncogene homolog B1) V600E or V600K mutations (18). Trametinib inhibits the growth of canine melanoma cell lines *in vitro* and in mouse models, accompanied by down modulation of phosphorylated extracellular signal-regulated kinase (ERK) accompanied by induction of apoptosis and cell cycle arrest (13, 19). Analogous effects on ERK activation and cytoproliferation due to trametinib have also been observed in canine bladder cancer organoids and tumor xenografts in mice (10).

In this study, combined inhibitor tolerability and plasma pharmacokinetics was examined, compared to single agents, in healthy laboratory dogs during short-term repeat dose oral administration. Effort was directed to determining a rational initial dose for treating canine naturally occurring cancers with elevated activities of one or both MAPK and PI3K/Akt cell signaling pathways and for future translation in the clinic. The aim is to provide beneficial therapeutic relief for dogs and eventually humans with mucosal melanoma.

## Materials and methods

### Animals, experimental design, and monitoring

Laboratory Beagle dogs (Charles River Labs, Mattawan, MI) served as surrogates in testing compounds proposed for application in canine clinical cancer care. The animals were at least six months old and weighed 6.4–8.8 kg at the initiation of study. They had routine veterinary care and had not been used in any previous studies. The study was conducted in AAALAC-accredited animal facilities [Charles River Laboratories, Inc., Mattawan, MI; NIH Office of Laboratory Animal Welfare Assurance approval (https://olaw.nih.gov/assured/app/index.html)], under review, monitoring, and approval of the facility's Institutional Animal Care and Use Committee according to U.S. Public Health Service national guidelines and regulations for the care and use of laboratory animals. The study was not designed to incorporate Good Laboratory Practices (GLP) regulatory criteria. Animals on study were monitored daily or more frequently and body weights were recorded, while periodic specimens were obtained for laboratory analyses. Clinical pathology analyses were included for hematology, serum chemistry, coagulation, urinalysis, and chemical bioanalysis was accomplished for pharmacokinetics (PK) (Table 1, repeat dose study) (Charles River Laboratories, Mattawan, MI).

**Table 1:** Treatment and sampling schedule for dogs given trametinib, sapanisertib or both in combination.

**Days**	**Group, 4 dogs each**			**Plasma, bioanalysis**			**Clinical pathology**			
	**1**	**2**	**3**	**once^#^**	0–24 h^*^	0–72 h^**^	**Hematology**	**Coagulation**	**Clinical chemistry**	**Urinalysis**	
−7				x			x	x	x	x	
1	T	S	TS			x					
2											
3											
4	T	S	TS								
5	T		T								
6	T	S	TS		x		x	x			
7	T		T				x	x	x		
8	T	S	TS								
9	T		T								
10	T	S	TS	x							
11	T		T				x	x			
12	T	S	TS								
13	T		T								
14	T	S	TS								
15	T		T	x			x	x			
16	T	S	TS								
17	T		T								
18	T	S	TS								
19	T		T								
20	T	S	TS			x	x	x	x		
21											
22											
23											
24											
25							x	x	x	x	
26											

# 2 h post dosing, on days 10 and 15.

* Pre-dose, and post dose 1, 2, 4, 8, and 24 h.

** Pre-dose, and post dose 1, 2, 4, 8, 24, 48, and 72 h.

T, trametinib; S, sapanisertib; TS, combination treatment.

Determining a 0.1 mg/kg sapanisertib dosage for repeat dose studies was based upon extrapolation of previous studies in mice (13, 20), leading to initial evaluation of escalating individual oral doses of 0.1, 0.5, 0.65, and 0.8 mg/kg in one male (M), and one female (F) dog with a ≥3-day washout period between doses. Dose selection for trametinib was based upon publicly available information provided to regulatory agencies for therapeutic approvals by the originator (Novartis) (21).

Six male and six female beagle dogs were enrolled in a parallel, three-arm, fixed, repeat oral-dose study. In Group 1, two male (22001, 22002) and two female (22501, 22502) dogs were given trametinib alone (0.025 mg/kg) daily (p.o., q.d.). In Group 2, two male (23001, 23002) and two female (23501, 23502) dogs were given sapanisertib alone (0.1 mg/kg) every other day (p.o., QOD). In Group 3, two male (24001, 24002) and two female (24501, 24502) dogs were given the combination of 0.025 mg/kg trametinib p.o., q.d. and 0.1 mg/kg sapanisertib p.o. QOD. All dogs were dosed on study day 1 to obtain single dose PK samples, and then dosed serially days 4–20. Dogs were fasted overnight prior to dosing and fed ad libitum 2 h (hrs) following dose administration. All animals were observed for 6 additional days (days 21–26) when dosing was completed, following which, dogs were returned to the general animal colony (Charles River Laboratories, Mattawan, MI).

Plasma samples for PK were collected at pre-dose (-7 days), then beginning day 1 following the initial dose of each study group at 1, 2, 4, 8, 24, 48, and 72 h. On day 6, plasma samples were collected pre-dose, then 1, 2, 4, 8, and 24 h post-dose. On days 10 and 15, a 2-h post-dose sample was collected. On day 20, plasma samples were drawn at pre-dose and 1, 2, 4, 8, 24, 48, and 72 h post-dose. Treatment schedule and clinical pathology specimen sampling intervals are outlined in Table 1.

### Small molecule inhibitor drugs

The drugs were supplied in capsule form (Wedgewood Pharmacy, Swedesboro, NJ). Both drugs were stored between 36 to 46°F and protected from moisture and light to support stability. Brief exposure to higher temperatures (<24 h) is considered acceptable. The pharmacy verifies composition and potency through independent third-party analyses and sets standard for variance on measured ingredients at +/- 5%, a range more limited than general US Pharmacopeia-recommended potency margins (+/- 10%). Neither drug has been approved for use in veterinary medicine. However, trametinib has shown promise against canine histiocytic sarcoma and canine urothelial bladder cancer *in vitro* and in mouse models (10, 22). Graphic depiction of inhibitor targeting within the cellular signal transduction pathway cascades is shown (Supplementary Figure S1).

### Assays for trametinib dimethyl sulfoxide (trametinib) and sapanisertib

Trametinib and sapanisertib were quantified in dog plasma collected using venipuncture, into blood tubes containing K_2_ ethylenediamine tetraacetic acid (EDTA) (BioIVT, Hicksville, New York), separated by centrifugation and stored frozen −80°C until analyzed in the laboratories of Charles River Labs (Mattawan, MI). Each drug analyte was measured in a total of 192 samples from 8 dogs. Propranolol hydrochloride, which has similar polarity and does not interfere with chromatography, served as an internal standard for each independent assay. Each 50 μL aliquot of known concentration standards, quality control sample, or study sample was mixed with 200 μL of working internal standard solution (10 ng/mL in acetonitrile). The samples were vortexed and centrifuged. A 150 μL aliquot of the resulting supernatant was transferred to a clean 96-well plate, evaporated, and reconstituted with 100 μL of water/acetonitrile (40/60, v/v). An aliquot was injected onto an LC-MS/MS XBridge BEH C18 liquid chromatography system (Waters Associates, Framingham, MA) having 50 x 2.1 mm (2.5 μm particle size) column with an isocratic flow consisting of water/formic acid (100/0.1, v/v) and acetonitrile/formic acid (100/0.1, v/v) at a flow rate of 0.3000 mL/min for analysis. The analyte and internal standard were detected using an API 6500+ triple quadrupole LC-MS/MS system (AB Sciex, LLC, Framingham, MA) equipped with an ESI (TurboIonSpray^®^) ionization source operated in the positive ion mode. Mass spectrometric Multiple Reaction Monitoring (MRM) transitions of the respective [M+H]^+^ ions were used to monitor trametinib (Table 2) and sapanisertib (Table 3).

**Table 2:** Mass spectrometric Multiple Reaction Monitoring (MRM) detection of trametinib.

	**Transition monitored**	**Retention time**
**Analyte**		
Trametinib:	*m/z* 616 → 491	0.9–1.1 min
**Internal standard**		
Propranolol:	*m/z* 260 → 116	0.5–0.6 min

*m/z*, mass divided by charge in atomic mass units; min, minutes.

**Table 3:** Mass spectrometric Multiple Reaction Monitoring (MRM) detection of sapanisertib.

	**Transition monitored**	**Retention time**
**Analyte**		
Sapanisertib:	*m/z* 310 → 268	0.4–0.5 min
**Internal standard**		
Propranolol:	*m/z* 260 → 116	0.4–0.5 min

*m/z*, mass divided by charge in atomic mass units; min, minutes.

Validation established for sapanisertib and trametinib standard calibration and quality control sample runs demonstrated that the analytical method was reproducible and linear across the range of 0.1–100 ng/ml, for each analyte. Linear regression analysis of the calibration curves indicated *R*^2^ values of 0.995 and 0.993 for sapanisertib and trametinib, respectively.

### Non-compartmental pharmacokinetic analyses

A non-compartmental approach to PK analysis was employed using Phoenix WinNonlin v8.3 (Certara Corp, Cary, NC) that was validated per FDA 21CFR Part 11 regulations. The maximum plasma concentration (C_MAX_) and the time of maximum plasma concentration (T_MAX_) were recorded as observed values. The area under the concentration-time curve (AUC) from time zero to the time of the final quantifiable sample (AUC_last_) was calculated using the linear-up/log-down trapezoidal method [model type Plasma (200-202)]. AUC_INF_ (the AUC from time zero to infinity) was calculated by extrapolation by dividing C_LAST_ (the last measurable drug concentration) by the rate constant of the terminal phase, λ_Z_. This constant was determined from the slope of the terminal phase of the concentration-time curve using uniformly-weighted least-squares as the estimation procedure and acceptance criteria of (i) adjusted *r*^2^ > 0.8, (ii) includes > 3 time points in the terminal phase occurring after the T_MAX_.

We estimated certain first dose PK parameters to include the apparent oral volume of distribution during the terminal phase (Vz/F) and the apparent oral systemic clearance (CL/F), which was calculated as absolute dose divided by AUC_INF_. If the extrapolated AUC_INF_ amount exceeded >25%, then the clearance estimates for those subjects were flagged and excluded from statistical summaries. This served as a reasonable standard nomenclature for reporting estimated clearance and volume as CL/F and V/F, as bioavailability (F) was not assessed in this study in the absence of intravenous dosing. For steady-state dosing, AUC_TAU_ (during the steady-state dosing interval) was used, as was apparent oral clearance at steady-state (CLssF; calculated as dose/AUC_TAU_). The accumulation index (AI) was estimated by dividing AUC_TAU_ at steady-state by AUC_LAST_ (over that dosing interval) following the first dose.

### Statistical analyses and characterization of PK

All summary statistics for these log-normally distributed PK data are presented as the arithmetic mean (average), median, standard deviation, %CV, and geometric mean (GM). To assess the extent of a drug interaction, for selected PK parameters (C_MAX_, AUC), a geometric mean ratio (GMR) was formed from the differences in each exposure metric in the combination treatment group relative to the single agent groups, along with a 90% confidence interval (CI). If this 90% CI exceeded the default *no effect* boundaries of 0.8–1.25 threshold, then we interpreted this to indicate a likely clinically significant change in that parameter, i.e. a drug-drug interaction. Between sex comparisons of C_MAX_ and AUC within treatment groups, and for an analyte across single agent and combination groups, were made using non-parametric two sample *T*-test (Mann-Whitney, alpha = 0.05;) (GraphPad Prism). Similar testing was conducted for comparing mean sample day values of activated partial thromboplastin time (APTT) in treated animals to those prior to drug administration for each group (MSExcel;v16.61.1).

## Results

### Trametinib dose demonstration

**Figure 1:**
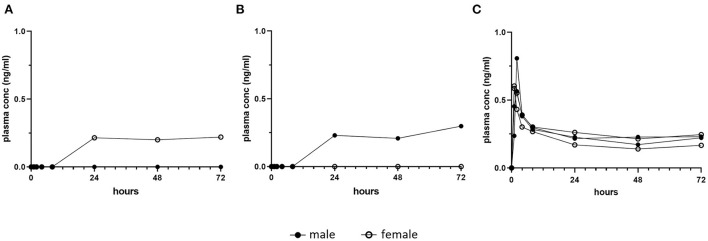
Pilot single dose pharmacokinetic plasma concentrations of trametinib in dogs. Healthy dogs (1 male, closed symbol and 1 female, open symbol) were fasted and treated orally with one dose of **(A)** 0.06 mg/kg trametinib, or **(B)** 0.03 mg/kg trametinib, as initially formulated. Detection in plasma was delayed until a minimum of 24 h post dose, and for each dose, one of two dogs remained below the level of assay detection (0.1 ng/ml). This inconsistency contrasts with **(C)**, trametinib plasma concentration in four fasted dogs given single oral dose of 0.025 mg/kg trametinib dimethyl sulfoxide (adapted from Figure 3A, initial day 1 dose, 0–72 h).

In a preliminary proof of principle dose and assay validation study, bioanalysis results of single oral dose plasma trametinib concentrations attained in four naive dogs were inconsistent and frequently below the assay detection limit, 0.1 ng/ml (Charles River Labs, Mattawan, MI) (Figure 1). These analytical findings, even under animal fasting conditions, were independently verified at the NCI Clinical Pharmacology Program. This result was interpreted to indicate that the trametinib formulation initially dispensed suffered from poor absorption in laboratory dogs. Consequently, trametinib was reformulated for this study by sulfation with equimolar dimethyl sulfoxide (DMSO) (Wedgewood Pharmacy, Swedesboro, NJ), consistent with the human formulation (MEKINIST^®^, Novartis), and used for all subsequent evaluations.

### Dose escalation tolerability of sapanisertib

**Figure 2:**
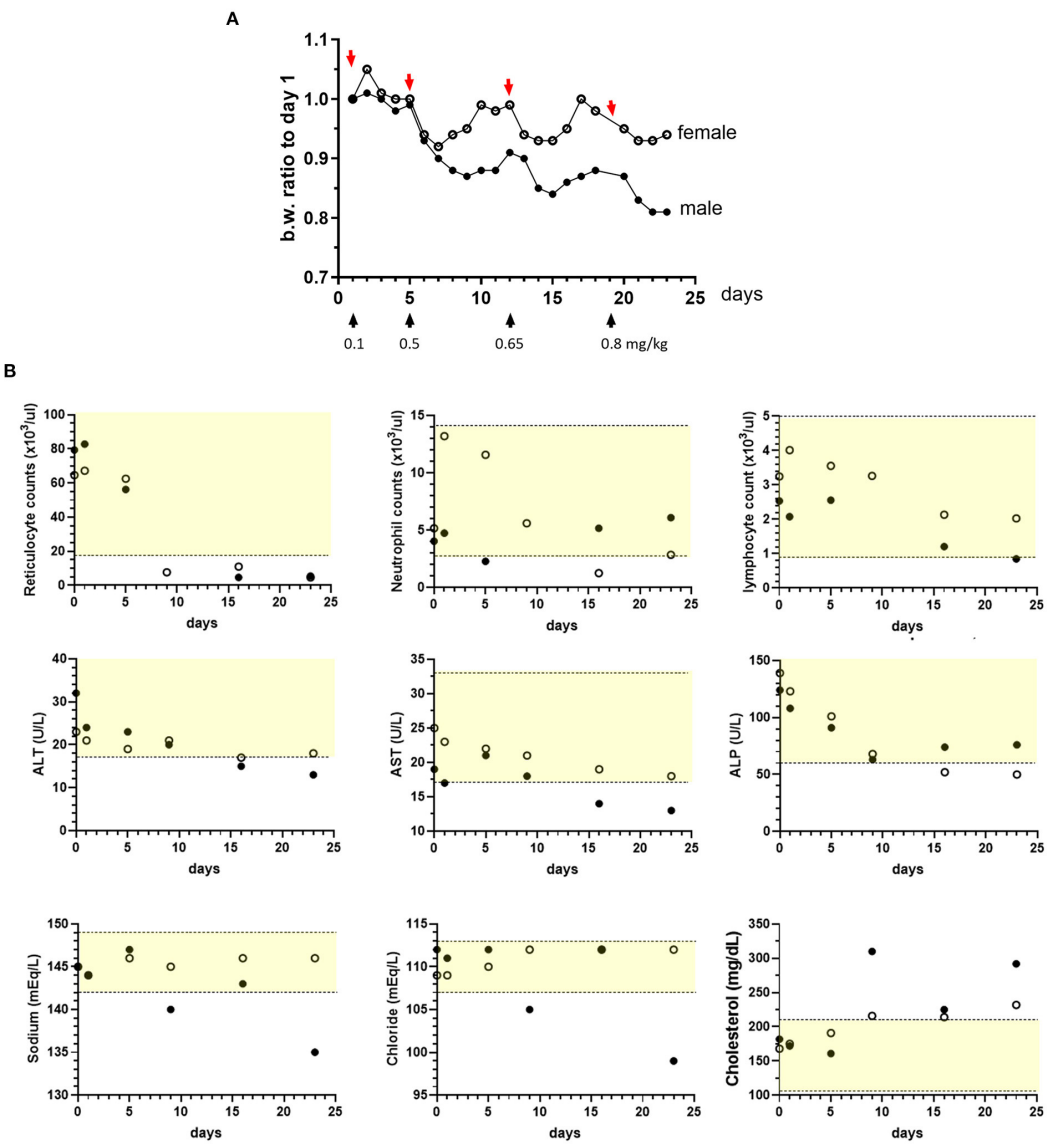
Sapanisertib escalating oral dose tolerability assessment in dogs. Two dogs [1 male, (filled circles) and 1 female, (open circles)] were treated with incrementally increased sapanisertib doses on day 1 (0.1 mg/kg), day 6 (0.5 mg/kg), day 12 (0.65 mg/kg) and day 19 (0.8 mg/kg). **(A)** Body weight (b.w.) changes with respect to time of first dose, in response to dosing as indicated. **(B)** Sapanisertib impact on select clinical pathology parameters for the testing days shown on x axis. Values on the y axis represent pre-treatment samples (0 days). Yellow-shaded area indicates reference range of values for normal healthy beagle dogs (Charles River Labs). Serum enzymes: ALT, alanine aminotransferase; AST, aspartate aminotransferase; ALP, alkaline phosphatase.

The initial efforts with sapanisertib focused on dose selection for ultimate testing in combination with orally administered trametinib dimethyl sulfoxide (trametinib) in nontumor-bearing laboratory beagle dogs. While a trametinib dose was selected from canine non-clinical regulatory information prepared for human drug approval (MEKINIST^®^, Novartis), a rational starting sapanisertib dose and schedule was based on previous studies in mice (see also materials and methods), as well as a pilot escalation trial in two dogs using sapanisertib alone. Sapanisertib dose escalation consisted of increased individual doses beginning with 0.10 mg/kg sapanisertib followed by a washout period of at least 3 days, and up to 7 days, between doses (Figure 2). Dogs lost body weight when dosed with ≥0.5 mg/kg sapanisertib. By day 23, the male dog had lost approximately 19% of initial body weight, while the female dog lost 7% body weight, and sapanisertib exposure was discontinued (Figure 2A). There was a trend toward recovery of modest amounts of body weight in these two dogs with the passage of sufficient time, post dose.

Following administration of ≥ 0.5 mg/kg sapanisertib, both sexes had moderate to marked decreases in red blood cell reticulocyte counts, minimal to moderate decreases in serum alkaline phosphatase (ALP) [as low as 0.36x (times) the pre-study value], aspartate aminotransferase (AST) (as low as 0.68x), and alanine aminotransferase (ALT) (as low as 0.41x), and minimal to mild increases in serum cholesterol concentrations (Figure 2B). Following administration of ≥ 0.65 mg/kg, both sexes also had mild to moderate progressive decreases in peripheral blood lymphocyte counts (as low as 0.33x), the female had mild to moderate decreases in neutrophil counts (as low as 0.24x), and the male had mild increases in globulin concentrations (up to 1.33x, globulin, not shown). Following administration of 0.8 mg/kg sapanisertib, the male also had mildly decreased sodium (0.98x) and chloride (0.89x) serum concentrations that may have been related to an episode of vomiting (Figure 2B). Selection of 0.1 mg/kg sapanisertib to initiate the follow-on combination repeat dose study was guided by these findings.

### Pharmacokinetic parameters in initial dose and repeat dose studies

Groups of 4 dogs (2 F, 2 M) were administered either single agents, or two-drug combination. Plasma concentration of either drug was measured over time following initial single oral doses (Day 1) as well as during repeat dosing, days 4-20 (Table 1). Trametinib was dosed at 0.025 mg/kg daily (q.d.) and sapanisertib was given at 0.1 mg/kg every other day (QOD). C_MAX_, T_MAX_, AUC, and T_1/2_ are indicated in Tables 4, 5 for trametinib and sapanisertib, respectively. C_MAX_ or AUC did not significantly differ between male and female dogs for either drug (p > 0.05).

**Table 4:** Non-compartmental analysis of trametinib with and without sapanisertib.

**Parameter**	**Trametinib (*n =* 4)**	**Trametinib + Sapanisertib (*n =* 4)**	**GMR (90%CI)^f^**
**First dose (Day 1)**			
C_MAX_ (ng/mL)	0.64 (18%)	0.79 (48%)	1.13 (0.79–1.63)
T_MAX_ (hr)^a^	1.5 (1.0–2.0)	1.5 (1.0–2.0)	n/e
AUC_0 − 24hr_ (hr*ng/mL)^b^	16.8 (14%)	16.7 (24%)	0.98 (0.75–1.29)
T_1/2_ (hr)	n/e^c^	149 (2.4%)^e^	n/e
**Steady-state (Day 6)**			
C_MAX_ (ng/mL)	2.18 (33%)	1.94 (60%)	n/e
**Steady-state (Day 10)**			
C_MAX_ (ng/mL)	2.89 (14%)	3.84 (40%)	n/e
**Steady-state (Day 20)**			
C_MAX_ (ng/mL)	4.19 (23%)	4.26 (40%)	0.96 (0.65–1.43)
T_MAX_ (hr)^a^	2.0 (2.0–4.0)	2.0 (1.0–8.0)	n/e
AUC_TAU_ (hr^*^ng/mL)	68.9 (20%)	74.5 (26%)	1.06 (0.82–1.38)
T_1/2_ (hr)	58.8 (53%)^d^	41.1(9.7%)	n/e

* Data presented as arithmetic means (%CV).

n/e: not evaluable, due to low numbers (n = 4) in each group.

a T_MAX_ reported as median (range).

b AUC_INF_ could not be calculated in this study due to extrapolation beyond the last time point exceeding 25%. Thus, CL/F and V/F could also not be calculated.

c All 4 subjects in this group had adjusted r^2^ <0.8, thus their half-life values cannot be trusted as accurate.

d One subject had an adjusted r^2^ <0.8, and was excluded from this summary.

e Two subjects had an adjusted r^2^ <0.8, and was excluded from this summary.

f 90% Confidence Intervals (CI) default no effect boundaries established with 0.8–1.25 threshold; the geometric mean ratio (GMR) values approximated 1.0, and few numbers of animals appears to have contributed to relative breadth of CI values.

**Table 5:** Non-compartmental analysis of sapanisertib with and without trametinib.

**Parameter**	**Sapanisertib (*n =* 4)**	**Sapanisertib + Trametinib (*n =* 4)**	**GMR (90%CI)^b^**
**First dose (Day 1)**			
C_MAX_ (ng/mL)	26.3 (21%)	21.9 (18%)	0.84 (0.74–0.94)
T_MAX_ (hr)^a^	2.0 (1.0–2.0)	2.0 (1.0–2.0)	n/e
AUC_INF_ (hr^*^ng/mL)	247.5 (41%)	192.4 (41%)	0.78 (0.48–1.28)
T_1/2_ (hr)	5.94 (15%)	6.03 (8.0%)	n/e
CL/F (L/hr/kg)	0.47 (51%)	0.59 (41%)	n/e
V/F (L/kg)	3.85 (34%)	5.04 (34%)	n/e
**Steady-state (Day 6)**			
C_MAX_ (ng/mL)	28.1 (14%)	18.1 (18%)	n/e
**Steady-state (Day 10)**			
C_MAX_ (ng/mL)	23.6 (22%)	19.3 (8.8%)	n/e
**Steady-state (Day 20)**			
C_MAX_ (ng/mL)	27.4 (22%)	20.2 (17%)	0.74 (0.55–0.99)
T_MAX_ (hr)^a^	2.0 (1.0–2.0)	1.5 (1.0–4.0)	n/e
AUC_TAU_ (hr*ng/mL)	249.8 (42%)	165.7 (32%)	0.68 (0.44–1.06)
T_1/2_ (hr)	6.28 (25%)	5.59 (14%)	n/e
CLss/F (L/hr/kg)^⊥^	0.46 (42%)	0.66 (35%)	n/e
V/F (L/kg)	3.97 (33%)	5.11 (21%)	n/e

* Data presented as arithmetic means (%CV).

n/e: not evaluable, due to low numbers (n = 4) in each group.

a T_MAX_ reported as median (range).

⊥ CLss/F (clearance at steady-state) calculated as Dose/AUCtau.

b 90% Confidence Intervals (CI) default no effect boundaries established with 0.8–1.25 threshold; geometric mean ratio (GMR) values skewed to the limits or exceeded 90% CI, while few numbers of animals appears to have contributed to relative breadth of CI values.

**Figure 3:**
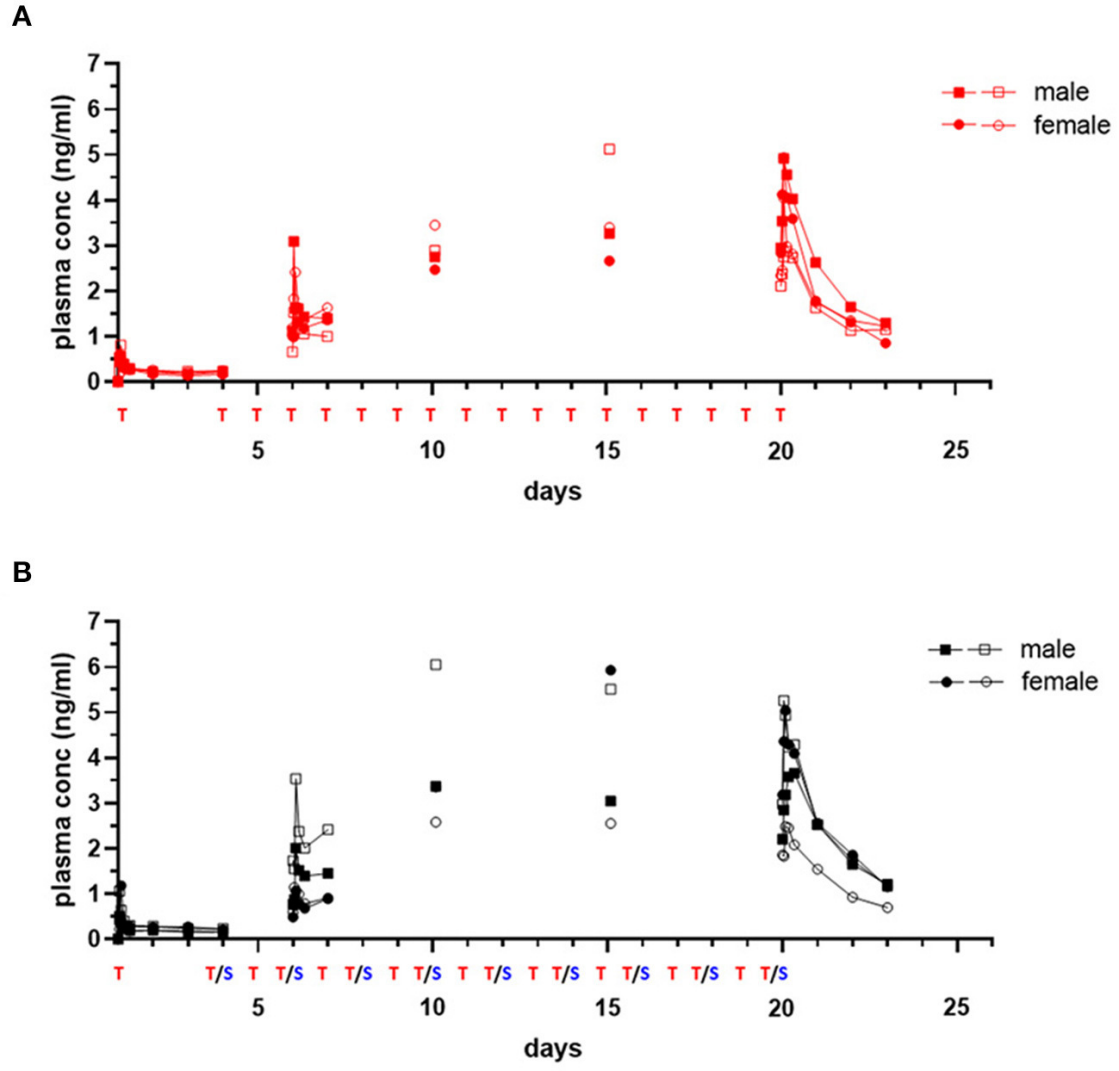
Single and repeat dose pharmacokinetic plasma concentration of oral trametinib in healthy dogs. Plasma values from two males, (square symbols) and two females (circle symbols) per group treated with trametinib alone [**(A)**, red tracing] or in combination with sapanisertib [**(B)**, black tracing] on day 1 and days 4–20 are shown. Treatment (T, trametinib; T/S, combination of trametinib and sapanisertib) schedule over time is labeled on X axis. The plasma trametinib concentrations are displayed from a 72-h PK post treatment series on days 1 and 20, a 24-h PK post treatment series on day 6, and PK single time samples on days 10 and 15 at 2 h after the treatment was administered.

T_MAX_ following initial dosing was similar comparing each drug given alone or in combination (Tables 4, 5). Plasma levels of trametinib revealed evidence of bioaccumulation (3-to-4-fold increase) from day 1 to day 20, for both Group 1, when given as single agent (Figure 3A), and Group 3, the two-drug combination (Figure 3B). The long half-life of trametinib was evident as well, although many subjects' estimates could not be used due to insufficient correlations (*r*^2^ <0.8) (Table 4).

A critical question regarding potential impact on trametinib drug concentrations resulting from the combination with sapanisertib, compared to trametinib alone, was answered by contrasting both first dose (day 1) and steady-state (day 20) exposure metrics (both C_MAX_ and AUC) according to geometric mean ratio (GMR) between the two treatments (Groups 1 and 3) with a 90% confidence interval (CI). Although the 90% CI exceeded the default *no effect* threshold of 0.8 – 1.25 for trametinib, the GMR generally approximated 1.0 (Table 4), suggesting sapanisertib had limited to no effect on trametinib PK. It is likely that the wide 90% CI represents variability among few numbers of samples (*n* = 4).

**Figure 4:**
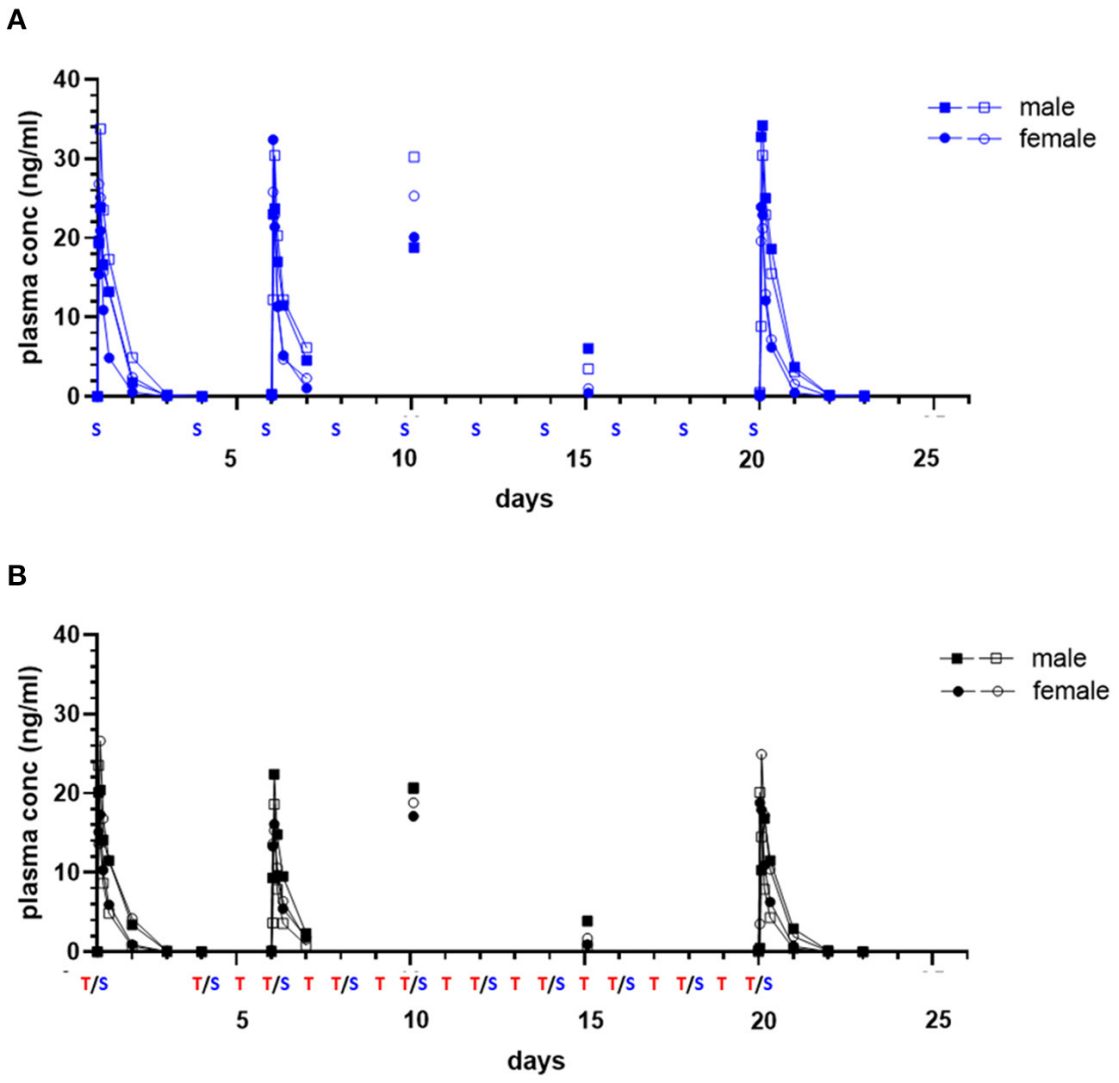
Single and repeat dose pharmacokinetic plasma concentration of oral sapanisertib in healthy dogs. Plasma values from two males, (square symbols) and two females (circle symbols) per group treated with sapanisertib alone [**(A)**, blue tracing] or in combination with trametinib [**(B)**, black tracing] on day 1 and days 4–20 are shown. Treatment (S, sapanisertib; T/S, combination of trametinib and sapanisertib) schedule over time is labeled on X axis. The plasma sapanisertib concentrations are displayed from a 72-h PK post treatment series on days 1 and 20, a 24-h PK post treatment series on day 6, and a PK single time samples on days 10 and 15 at 2 h after a treatment was administered.

Initial and repeat dose sapanisertib was rapidly absorbed (range 1–4 h) (Table 5). In contrast to trametinib, sapanisertib was not observed to significantly accumulate with repeat dosing from day 1 to day 20 due to a much shorter half-life (range 4.8–6.8 h) (Figure 4; Table 5). This is further supported by the plasma levels at day 15, wherein minimal detectable sapanisertib is observed approximately 24 h from the previous dose (Groups 2,3) (Figure 4). Moreover, in contrast to the lack of apparent sapanisertib interaction on trametinib levels noted above, sapanisertib plasma concentration was influenced when combined with trametinib. On day 1, both sapanisertib C_MAX_ and AUC (AUC_INF_) were significantly lower in the combination arm (Group 3) vs. sapanisertib alone (Group 2), by a similar magnitude (GMR, 0.84, 0.78, for C_MAX_ and AUC_INF_, respectively) (Table 5). This finding is further supported by the exaggeration of this effect at steady-state; on day 20, the GMR for sapanisertib C_MAX_ and AUC (AUC_TAU_) were 0.74 and 0.68, respectively. Half-life and T_MAX_ were largely unchanged over time and between arms (Table 5).

### Repeat dose tolerability as assessed by clinical observations and hematology parameters

**Figure 5:**
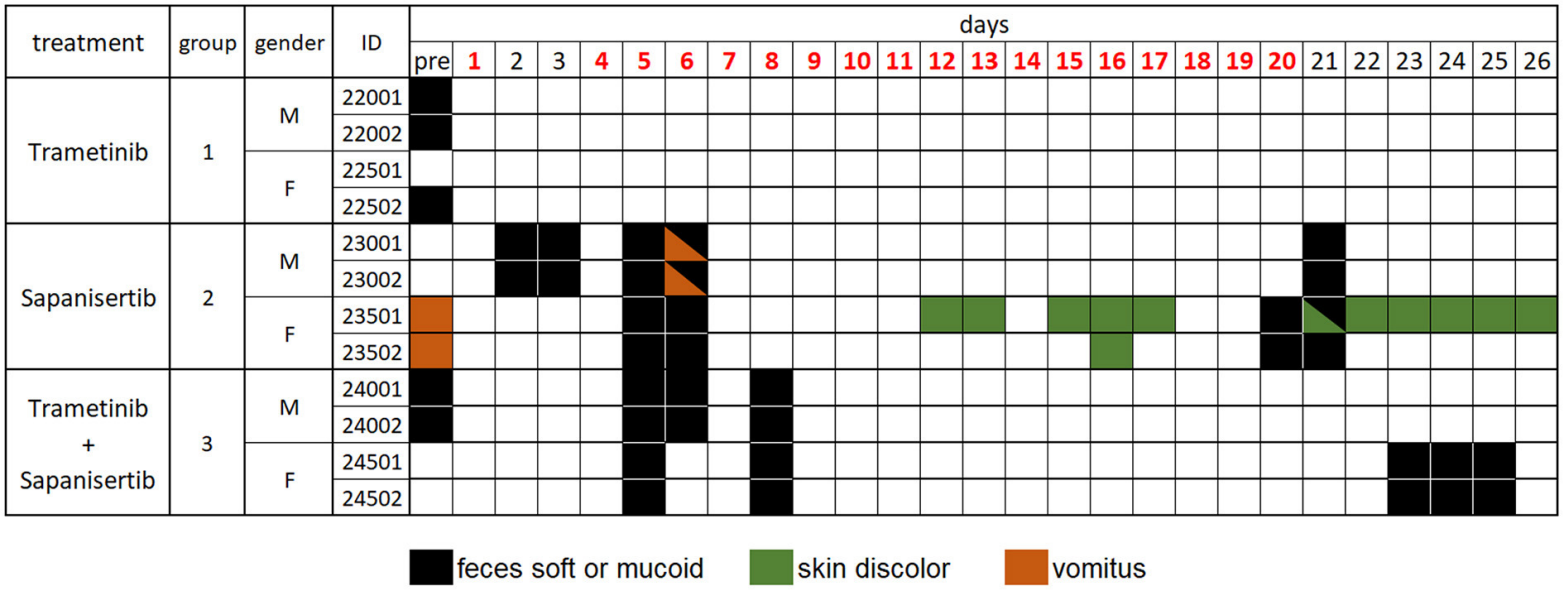
Daily individual record of observations for tolerability and adverse events in treated dogs, by treatment group over time. Days shown in red font indicate treatment given. Observations were generally considered treatment-related, except for those recorded as “pre”, prior to initiation of treatment, and were otherwise largely self-limiting episodes. Dog 22001 exhibited discolored teeth and dog 24501 displayed signs of estrus once the study was initiated. Animal identification numbers and sex (M, male; F, female) are indicated along with treatment group.

**Figure 6:**
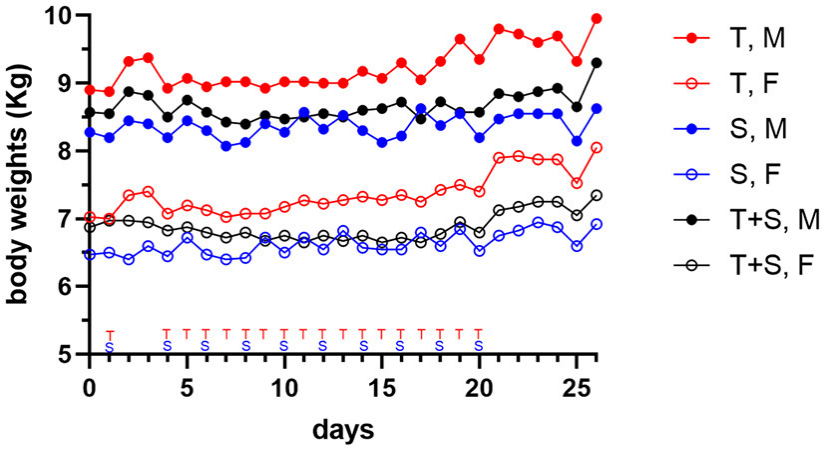
Mean body weight changes in dogs (two male (M) closed circle, or two female (F), open circle, per group) treated orally with trametinib (T, red tracing), sapanisertib (S, blue tracing), or combined trametinib and sapanisertib (T+S, black tracing), at times indicated on the X-axis.

Dogs treated by repeat-dosing for up to 17 days did not experience serious adverse events or dose limiting toxicities. Treatment-associated clinical signs included transient episodes of soft or mucoid feces (*n* = 8), vomiting (*n* = 2), or skin discoloration (*n* = 2) (Figure 5). Side effects observed (grade 1,2) did not require intervention and were more often attributed to sapanisertib in single agent and combination treatment groups. One additional dog each exhibited either discolored teeth (22001) or signs of estrus (24501). No substantive impact on body weight was noted for either sex over the course of the repeat dose study due to any of the three treatment conditions (Figure 6).

**Figure 7:**
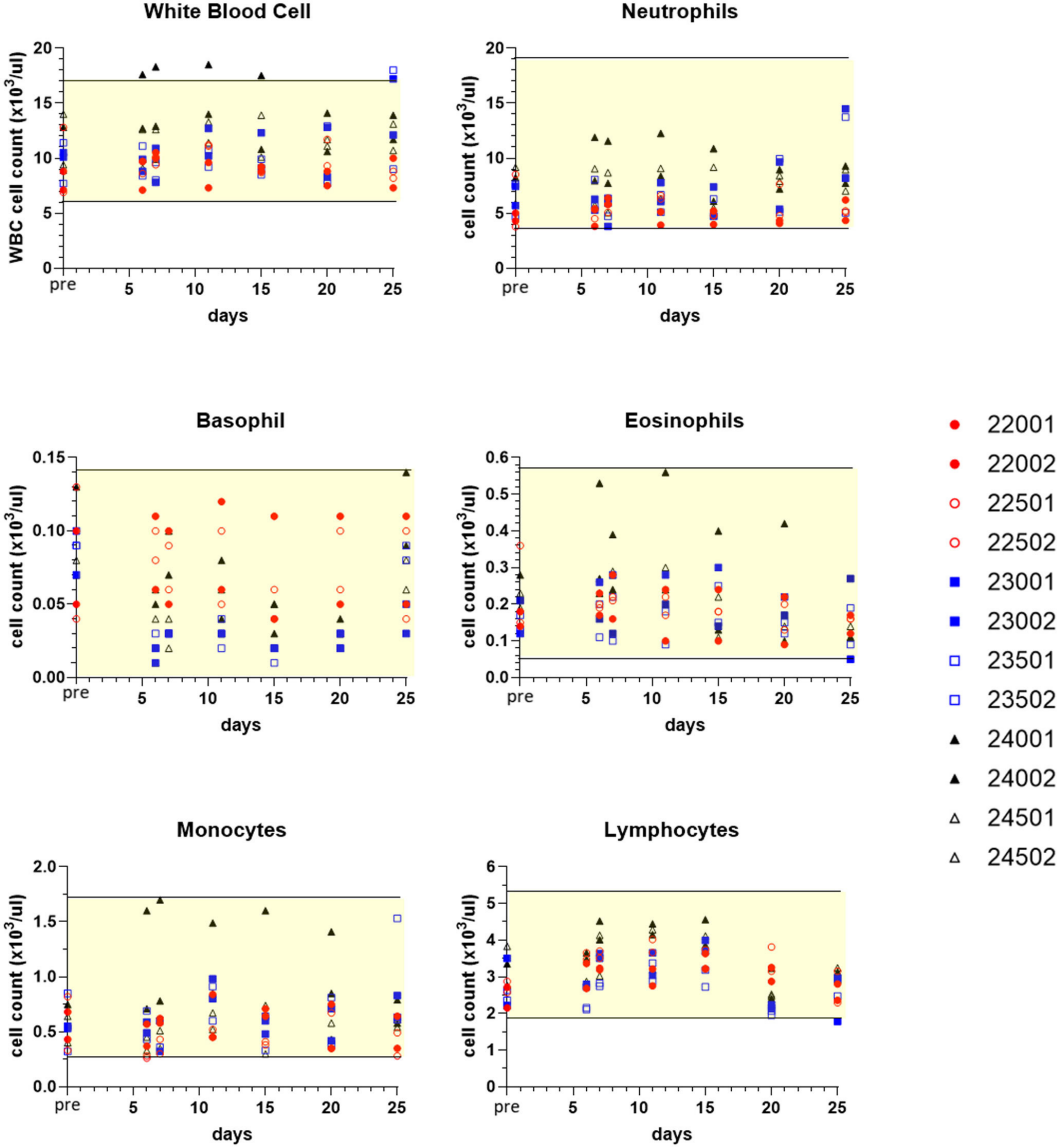
Selected white blood cell count values of peripheral blood leukograms for individual treated dogs over time. Blood samples were obtained 7 days before the first dose (pre, Y axis) and on study days 6, 7, 11, 15, 20, and following discontinuation of treatment on day 25 (serial treatment days 4–20). Yellow-shaded area indicates reference range of values for normal healthy beagle dogs (Charles River Labs). Dog identification numbers, sex and treatment observations for animals given trametinib (red symbols), sapanisertib (blue symbols), and combination treatment (black symbols) are referenced in Figure 5.

Sampling for hematology, serum chemistry, coagulation and urinalysis was conducted to facilitate monitoring treatment effects over various time points, and values obtained were compared to pre-treatment measurements for each subject (Table 1). Although generally modest, treatment altered some clinicopathological parameters. Three dogs experienced mildly increased circulating blood neutrophils (Figure 7). Neutrophils increased up to 1.95x individual pre-study values in one male (23002) and one female (23501) receiving sapanisertib only (Group 2) on days 20 and 25. One Group 3 male receiving the two-drug combination (24001) experienced up to 2.6x increased blood neutrophils days 6 through 25. Minimal fluctuations in values of other leukocyte populations appeared to return to pretreatment values (normal ranges) for most animals upon discontinuation of drug exposure (day 25) (Figure 7).

**Figure 8:**
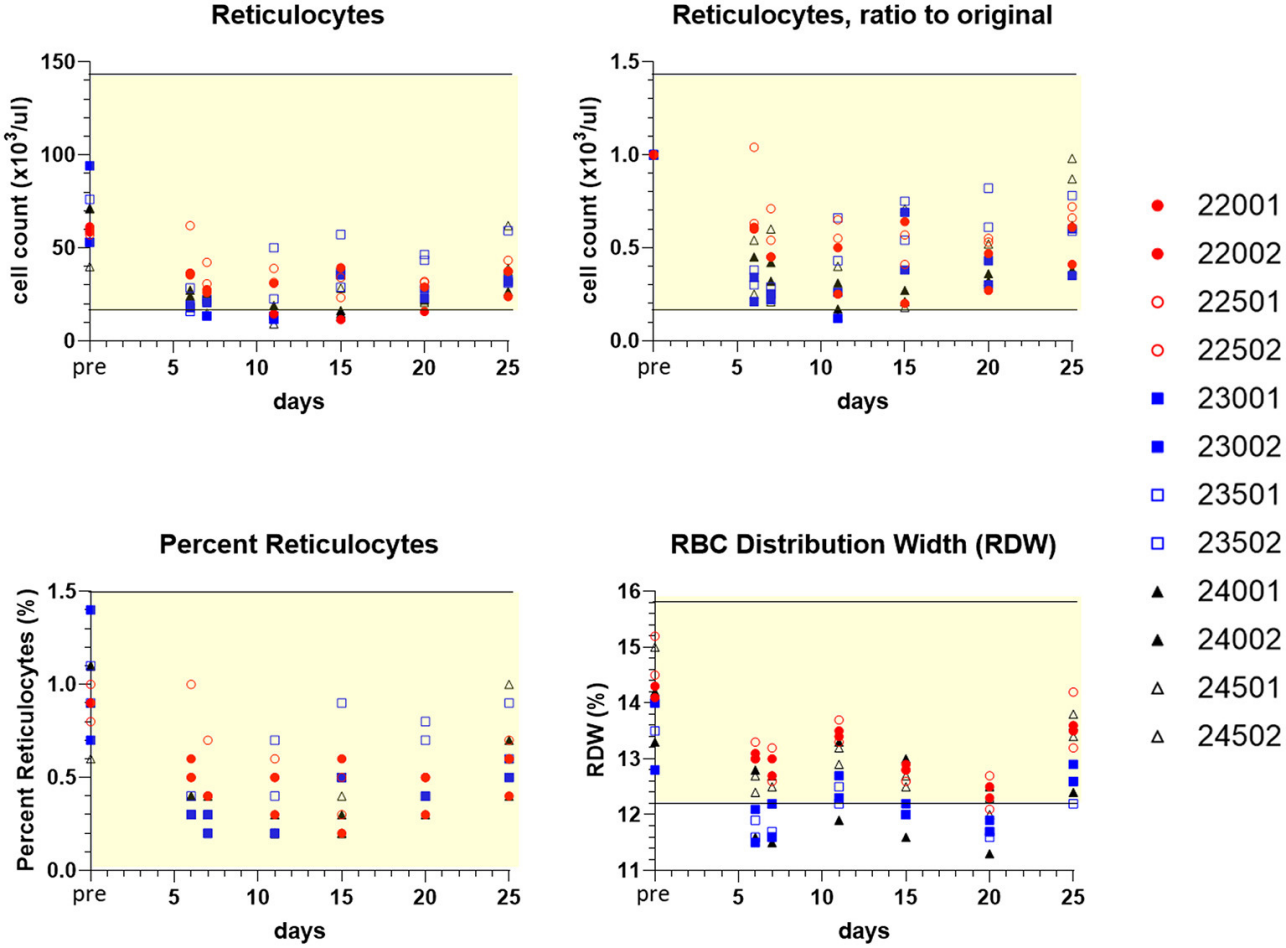
Values of peripheral blood reticulocytes and erythrocyte (RBC) distribution width (RDW) for individual treated dogs over time. Blood samples were obtained 7 days before the first dose (pre, Y axis) and on study days 6, 7, 11, 15, 20, and following discontinuation of treatment on day 25 (serial treatment days 4–20). Percent reticulocytes represents cell counts referenced to the total RBC count (Supplementary Figure S2). Yellow-shaded area indicates reference range of values for normal healthy beagle dogs (Charles River Labs). Dog identification numbers, sex and treatment observations for animals given trametinib (red symbols), sapanisertib (blue symbols), and combination treatment (black symbols) are referenced in Figure 5.

On days 6–25, both sexes in all treatment groups had minimal to marked decreases in individual absolute reticulocyte counts (as low as 0.12x) (Figure 8). Typically, this occurred with concomitant decreases in red cell distribution width (RDW), as low as 0.82x pretreatment value (indicative of decreased variability in red blood cell size). Additionally, 10 out of 12 dogs exhibited minimal to mild decreases in individual red blood cell mass parameters (erythrocyte count, hemoglobin concentration, and/or hematocrit; as low as 0.81x) from day 6 to day 25 (Supplementary Figure S2). Compared to day 20 at final dosing, day 25 sampling revealed a trend toward recovery of circulating reticulocytes (Figure 8), accompanied by greater reticulocyte corpuscular hemoglobin content (CHr) and mean reticulocyte corpuscular volume (MCVr) (data not shown). This was consistent with a release of larger young red blood cells in all treatment groups, once drug was discontinued.

Urine samples collected prior to treatment and at the conclusion of monitoring were analyzed. On Day 25, one male dog given trametinib only (22001), three of four dogs given sapanisertib only and two of four dogs treated with trametinib plus sapanisertib in combination had minimal to marked increases in urine protein concentration (greater than values recorded pre-study, up to ≥1,000 mg/dL measured semi-quantitatively by urine dipstick) (Supplementary Table S1). Additionally, individuals of both sexes in Groups 2 (sapanisertib only) and 3 (combination) had minimal to mild increases in urine glucose (up to 250 mg/dL measured semi-quantitatively, by urine dipstick). Group 2 males and both sexes in Group 3 also had moderate increases in urine pH (up to pH ≥ 9.0). These increases in urine protein, glucose, and pH were considered related to trametinib, sapanisertib, and/or trametinib plus sapanisertib administration.

Potential for the treatments to influence laboratory analysis of blood coagulation was also monitored. There were minimal to moderate increases at various time points in individual animal fibrinogen concentration (up to 2.61x), which were most pronounced in Group 3 samples, having received the combination (Supplementary Figure S3A). These increases in blood fibrinogen concentration were considered related to trametinib, sapanisertib, and combination trametinib plus sapanisertib administration. Compared to pretreatment samples, activated partial thromboplastin time (APTT) assay was prolonged up to 1.21x during treatment, from Days 6, 11, 15, 22, and/or 25 in one male and one female dog given sapanisertib only, and both males and one female dog treated with the two-drug combination (Supplementary Figure S3B). Further analysis of group mean APTT values for dogs receiving sapanisertib either alone or in combination revealed consistently significant differences beginning day 11 and later, compared to dogs receiving trametinib alone. In addition, these mean APTT values for dogs receiving sapanisertib alone or in combination were also notably distinct from pre-treatment mean values of all dogs. Despite this possible sapanisertib treatment influence, none of the analyzed APTT values from any treated dogs exceeded normal dog reference range upper limits and were not clinically relevant in this study (Supplementary Figure S3B). No analogous effect was seen for Prothrombin time (PT) assays.

Several additional minor fluctuations occurred among a variety of serum biochemical parameters during therapy. These minimal biochemical changes did not necessitate intervention. However, selected observations are summarized (Supplementary File 1) and may serve as possible hallmarks of therapeutic impacts that could be more profound in older dogs with spontaneous cancers and potential co-morbidities.

## Discussion

Combined targeting of MEK and mTOR has been successfully applied in several preclinical cancer model systems, leading to potential opportunities to overcome some forms of acquired drug resistance in liver and pancreatic cancers (23, 24). This precision targeting approach has included the synergistic parallel use of combined trametinib and sapanisertib specifically, in glioblastoma (25) and melanoma (13). To advance this development toward clinical application, our study employed daily trametinib and every other day sapanisertib as either single agents, or as a combination for 17 days in nontumor-bearing healthy laboratory dogs to inform therapy in the clinic for dogs with mucosal melanoma. These treatments in the parallel, fixed-dose, repeat-dose three arm study were relatively well tolerated, accomplished without significant body weight change, requirement to intervene clinically, or the need to interrupt drug administration during the short-term exposure. Treatment-related responses experienced by dogs included infrequent episodes of mostly self-limiting loose, mucoid feces and skin discoloration or vomitus, accompanied by alterations in clinical pathology parameters.

Developing sapanisertib in combination with trametinib in this canine study was based on a mouse model using an every-other-day schedule of administration (13), as well as our dose escalation described in this study. Sapanisertib continues under clinical investigational development through various doses and schedules for humans (17). Considering body weight loss, and fluctuations in hemograms and urinalyses that occurred at doses ≥0.1 mg/kg, the oral dose administered repeatedly appeared to be a most well tolerated level in dogs. Trametinib non-clinical dose and schedule data previously available for dogs (21) served to guide the daily oral administration of 0.025 mg/kg trametinib.

Treatments in all groups tended to diminish red blood cells and trigger an acute phase inflammatory reaction, the latter primarily characterized in blood samples by increased circulating neutrophils, increased fibrinogen, decreased serum albumin and increased globulin. Although hemogram and biochemical parameters varied from pre-treatment measurements in response to sapanisertib, and/or trametinib, the degrees of alteration were limited, rarely transitioning substantially outside normal healthy beagle dog reference range values, if at all. The most notable change in clinical laboratory values appeared to be the treatment effect on erythropoiesis. Observed previously in trametinib-treated dogs (EMA, European Public Assessment Report: MEKINIST^®^), decreased circulating reticulocytes and associated alterations in red blood cell indices in the present study occurred as a dynamic treatment response within all three groups; with apparent abatement of the effect beginning after day 20 upon withdrawal of the compounds. The changes are consistent with transient drug-induced erythroid production and maturation arrest. Subject to small molecule inhibition, both the MAPK and PI3K/Akt pathways are downstream of erythropoietin and transferrin receptor signaling, which play major roles in normal erythropoiesis (26, 27). At least under acute induction, decreased circulating reticulocytes may serve as a pharmacodynamic surrogate, amenable to routine blood sampling, indicating some level of host target inhibition in dogs at the doses tested. Moreover, this drug effect should be monitored during cancer therapy over time to determine if it may become a deleterious sequela that is uncompensated.

Proteinuria, glycosuria, and increased urine pH were most prominent in sapanisertib and combination treated dogs, and were interpreted to indicate renal effects mainly attributable to treatment with sapanisertib. In a preclinical model, acute kidney injury (AKI) with moderate histopathological cortical tubular epithelial degeneration and necrosis was observed in some mice given 2.5 mg/Kg sapanisertib daily (13). By contrast, mice treated every other day with the same dose of sapanisertib did not exhibit substantive AKI, and instead had isolated and discrete renal tubular cell death accompanied by individual tubular cell mitosis in tissue sections, indicative of low-grade cell turnover with compensatory regeneration. The 0.1 mg/kg sapanisertib dose used in dogs was considered less than the 2.5 mg/kg in mice (13), the latter of which would be approximately analogous to 0.37 mg/kg in dogs (20). Regardless, potential for occurrence of renal drug effects analogous to sapanisertib exposure in mice must be considered in treated dogs, particularly for patients with nephropathy.

Prolongations in APTT documented in some dogs, primarily in treatment groups 2 and 3, appeared to implicate possible sapanisertib as well as sapanisertib and trametinib combination drug effects. While vigilance is prudent, all prolonged APTT changes were minimal, and values obtained for treated animals fell well within the normal dog reference range (or below); no clinical intervention or drug dose adjustment was needed in this study. The duration of drug exposure was relatively short term however, and the potential for treatment to affect coagulation cascades in canine cancer patients, beyond minor influence on the *in vitro* assay, should be considered.

The trametinib PK parameters achieved in the current study, including C_MAX_ and AUC, closely replicated canine values reported previously to regulatory agencies (28). Likewise, the absence of significant differences in C_MAX_ and AUC values when comparing male to female dogs from the current study also mirrored these regulatory results. It is noteworthy that the limited withholding of food pre- and post-dosing, coupled with the sulfation of trametinib with DMSO, substantially contributed to measurable plasma levels, a likely consequence of improved oral absorption due to greater gastric solubility of the sulfated formulation. Furthermore, in approximation with the dose and schedule tested, trametinib has been tolerated by dogs for longer 13-week periods (28). In comparing trametinib PK parameters between dogs and humans, there was some correspondence, but not uniformly so. Known trametinib bioaccumulation in humans (29, 30) was observed in dog plasma, with both dogs and humans having up to six-fold accumulation upon repeat daily dosing. More rapid clearance of trametinib in dogs (0.37 L/hr/kg) compared to humans (0.07 L/hr/kg; MEKINIST^®^ Prescribing Information) in association with the wider distribution volume of trametinib in dogs (32 L/kg) relative to humans (3.1 L/kg), could possibly be due to differences in protein binding, as trametinib is 97% bound by human serum albumin. Overall, the systemic exposure (AUC_TAU_) of trametinib on day 20 (69 hr^*^ng/mL) following a 0.025 mg/kg daily regimen (an approximately 0.2 mg dose for an average 8 kg beagle dog) was about 1/3 that experienced by humans given a 2 mg dose (31). However, caution is warranted when making cross-species comparisons of PK, particularly for this study with only four canine subjects receiving trametinib alone.

Sapanisertib was rapidly absorbed by dogs (T_max_ 2 h on average) and did not display accumulation with repeated QOD dosing. The pharmacokinetic profile findings were similar to single-agent human studies (17, 32). While the presence of sapanisertib lacked substantial effect on trametinib PK when given in combination, the reverse proved otherwise. On day 1, both sapanisertib C_MAX_ and AUC (AUC_INF_) were significantly less in the combination arm vs. sapanisertib alone, a finding that was foreshadowed in a mouse model (13). In combination with trametinib, sapanisertib clearance in these Group 3 dogs was faster and distribution volume greater (lower exposure) compared to dogs in Group 2 treated with sapanisertib alone, outcomes that are consistent with a compensatory action for the lower AUC. Trametinib is a known inducer of cytochrome P450 CYP3A4 (33, 34). Thus, induction of cytochrome P450 is a likely mechanism behind the faster clearance and lower exposure (C_MAX_ and AUC) of sapanisertib during combination treatment, relative to sapanisertib alone. This is at least consistent as well with the additional accumulation of trametinib during repeat dosing and that being plausibly accompanied by further cytochrome P450 microsomal induction, which could therefore consequently be reflected in the observed continued sapanisertib diminution when combined with trametinib at the later time point. Unfortunately, published evidence to confirm the specific CYP isoform was not uncovered, but based on this dog study, CYP3A family induction appears to be a candidate metabolic mechanism.

Inter-species comparison of canine sapanisertib exposures following 0.1 mg/kg (roughly 0.55–0.85 mg for beagle dogs averaging 7.4 kg) to PK of human cancer patients receiving 4 mg oral sapanisertib, a dose level that has progressed into phase II trials, revealed correspondences (35). Mean animal to human exposure ratios (canine sapanisertib only group) were 1.11 for C_MAX_, 1.31 for AUC_INF_, and 0.91 for T_1/2_. Therefore, sapanisertib levels achieved in these dogs would presumptively have potential clinical relevance based upon this comparison to humans.

Evaluating this inhibitor combination in laboratory beagle dogs extends previous findings in mice (13), and serves as a necessary and beneficial step in the drug development process. Similarly, the corroboration of general conclusions across species bolsters the validity of the evidence obtained in a manner addressing scientific reproducibility, frequently a concern in preclinical studies (36). While a degree of overlap with our previous approaches would logically be expected, notable differences between this and the previous mouse study (13) are worth mention. Among these included specifically testing both a sulfated and non-sulfated formulation of trametinib in the current study, finding that the former was necessary in dogs whereas non-sulfated trametinib served similar purpose resulting in measurable PK analyses only in mice. Maximum tolerated doses were different for the two drugs between the two species and fasting prior to administration was not necessary in mice. A drug-drug interaction occurred during administration of the combination in both species, however the outcomes diverged between the studies. As noted, sapanisertib AUC in dogs was diminished in the presence of trametinib (mean GMR exceeded 90% CI), portended by finding a similar trend in mice; whereas in contrast, trametinib AUC was significantly increased singularly in mice only during combination treatment (p <0.001) (13). The current study was a survival study, in contrast to the efficacy endpoints possible with tissue collection in the melanoma xenograft mouse model, which led us to incorporate greater use of hematology and clinical chemistry to monitor treated non-tumor bearing dogs. Consequently, we realized the potential to further monitor circulating blood reticulocytes as a candidate pharmacodynamic biomarker in dogs. Collectively, these distinctions represent expanded parameters that can inform a more meaningful canine clinical trial in dogs with spontaneous cancer.

The approach extending preclinical findings in mice to laboratory studies in dogs was also designed to address evolving processes for obtaining institutional approvals of veterinary clinical trial proposals (37). For example, prior to clinical trial study proposal review, some institutional Veterinary Clinical Study Committee Review Boards increasingly expect preliminary evidence of reasonably safe administration *in the target species* during early dose range finding for agents intended for investigation in client-owned pets with naturally occurring cancer. Our approach also practically addresses recognized concerns about the eventual significant attrition of candidate therapeutics upon their investigation in the clinic, when extrapolations are made primarily from rodent testing during the drug development pipeline (38). Furthermore, evidence that we have developed in mice and dogs is analogous to expected non-clinical testing in multiple species prior to human exposure by regulators (39). Finally, additional justification for the iterative approach includes recognition that pharmaceutical companies rarely pursue drug development specifically for orphan disease entities, such as rare cancer subtypes. This may be also true where the drugs proposed for combinations have unique ownership, such that the typical new drug application filing and commercial return on investment in either case might logically be constrained. Consequently, public scientific investment can serve to fill gaps in important, challenging, and neglected problems in cancer research.

Overall, the findings developed in this repeat dose study support further development of these inhibitors in combination for clinical cancer care. The cumulative evidence from foregoing studies (11, 13, 15, 17, 19, 21) as well as data in the present study indicate the dose and schedule evaluated are an appropriate initial run-in for a clinical trial of dogs with spontaneous cancers driven with the relevant target activation. The relative tolerability and promising PK characteristics of the combination in dogs during the short-term repeat dose exposures support further evaluation of effectiveness in canine clinical trials for dogs with naturally occurring melanoma and other cancers with activation of one or more of the RAS/MAPK and PI3K/Akt/mTOR pathways. Evidence presented here also supports this combination as a translational comparative oncology opportunity worthy for informing possible treatment in humans with mucosal melanoma.

## Data availability statement

The raw data supporting the conclusions of this article will be made available by the authors, without undue reservation to qualified investigators for scientific inquiry.

## Ethics statement

The study was conducted in AAALAC-accredited animal facilities [Charles River Laboratories, Mattawan, MI; NIH Office of Laboratory Animal Welfare Assurance approval (https://olaw.nih.gov/assured/app/index.html)], under review, monitoring, and approval of the facility's Institutional Animal Care and Use Committee according to U.S. Public Health Service national guidelines and regulations for the care and use of laboratory animals.

## Author contributions

B-RW and RS conceived, designed, directed the study, analyzed and interpreted results, and wrote and approved the manuscript. CP, WR, and WF analyzed pharmacokinetic specimens and data and interpreted results and edited and approved the manuscript. SH contributed to study design, data analysis, interpreted results, and edited and approved the manuscript. All authors contributed to the article and approved the submitted version.
