# Supplementary material for: Pharmacokinetics and tolerability of the dual TORC1/2 inhibitor sapanisertib in combination with the MEK inhibitor trametinib in dogs
Source: Front Vet Sci. 2022 Dec 14;9:1056408. doi: 10.3389/fvets.2022.1056408 (PMC9794608; doi:10.3389/fvets.2022.1056408)
Supplement: Supplementary file 1 [file Data_Sheet_1.docx]

Wei, BR et al combination sapanisertib and trametinib PK in dogs

Supplemental results file

On Days 7, 20, and/or 25, one male dog given trametinib only (Animal No. 22002) and both sexes in the combination-treated group had minimal to mild decreases in albumin concentration (as low as 0.73x) and/or increases in globulin concentration (up to 1.45x), typically with concomitant decreases in albumin/globulin ratios (as low as 0.53x). These changes were considered related to trametinib and trametinib plus sapanisertib administration. Along with increases in neutrophil counts in a few trametinib only and sapanisertib only treated individuals, increases in blood fibrinogen concentration measured in animals from all groups were considered consistent with an acute phase inflammatory reaction.

On Days 7 and 20, both sexes in all groups had a trend for minimal to mild decreases in serum phosphorus concentrations (as low as 0.74x) that were considered related to trametinib, sapanisertib, and the combination. The decreased serum phosphorus concentrations in those affected had partially to fully resolved on Day 25. On Days 7 and 20, both sexes in Group 2 and males in Group 3 had minimal increases in cholesterol concentrations (up to 1.50x). These changes were considered related to sapanisertib (Group 2) and trametinib plus sapanisertib (Group 3) administration and had partially to fully resolved on Day 25.

On Day 20, one female dog given sapanisertib only (Animal No. 23502) had a minimal increase in serum ALT (2.10x). Additionally, on Day 20, one male dog given the combination (Animal No. 24001) had a minimal increase in serum AST (2.58x). However, due to the low magnitude as well as infrequent incidence of these findings, and lack of a consistent change between sexes or across intervals, these changes were considered unlikely to be small molecule inhibitor related. All other fluctuations among individual and mean clinical chemistry values were considered sporadic, consistent with biologic and/or procedure-related variation, and/or negligible in magnitude, and not related to trametinib, sapanisertib, or trametinib plus sapanisertib administration.
