# Supplementary material for: Pharmacokinetics and tolerability of the dual TORC1/2 inhibitor sapanisertib in combination with the MEK inhibitor trametinib in dogs
Source: Front Vet Sci. 2022 Dec 14;9:1056408. doi: 10.3389/fvets.2022.1056408 (PMC9794608; doi:10.3389/fvets.2022.1056408)
Supplement: Supplementary file 2 [file Data_Sheet_2.docx]

SUPPLEMENTARY FIGURES AND TABLE

Pharmacokinetics and tolerability of the dual TORC1/2 inhibitor sapanisertib

in combination with MEK inhibitor trametinib in dogs

Bih-Rong Wei^1,2^, Cody J. Peer^3^, William Richardson^3^, Stephen M. Hewitt^4^, William D. Figg^3,5^, and R. Mark Simpson^1*^

^1^Laboratory of Cancer Biology and Genetics, Center for Cancer Research, National Cancer Institute, Bethesda, Maryland, ^2^Leidos Biomedical Research, Inc., Frederick National Laboratory for Cancer Research, Frederick, Maryland, ^3^Clinical Pharmacology Program, Office of the Clinical Director, ^4^Laboratory of Pathology, and ^5^Genitourinary Malignancies Branch, Center for Cancer Research, National Cancer Institute, Bethesda, Maryland


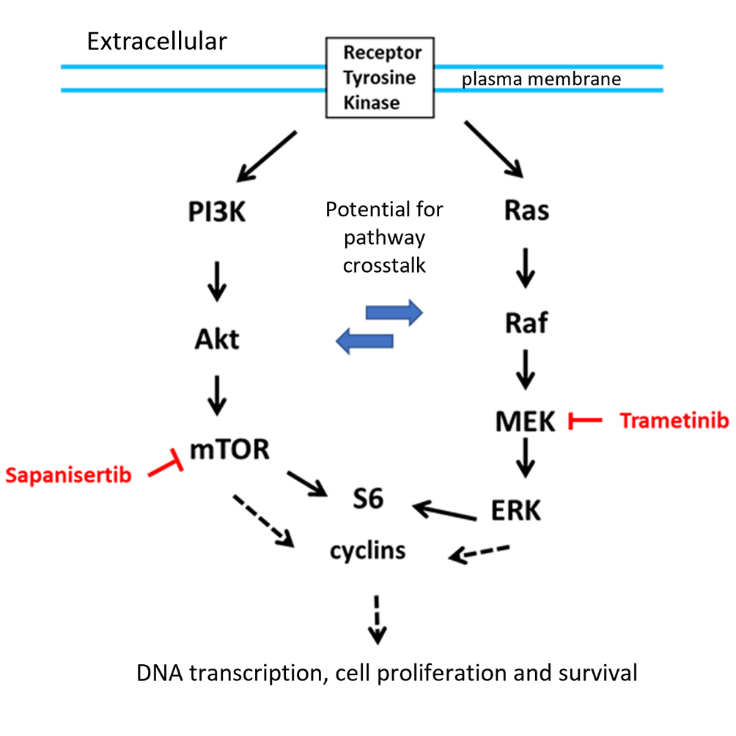


**Supplementary Figure S1**. Graphic depiction of example RAS/MAPK and PI3K/Akt/mTOR signal transduction pathways with indication of inhibitor target.


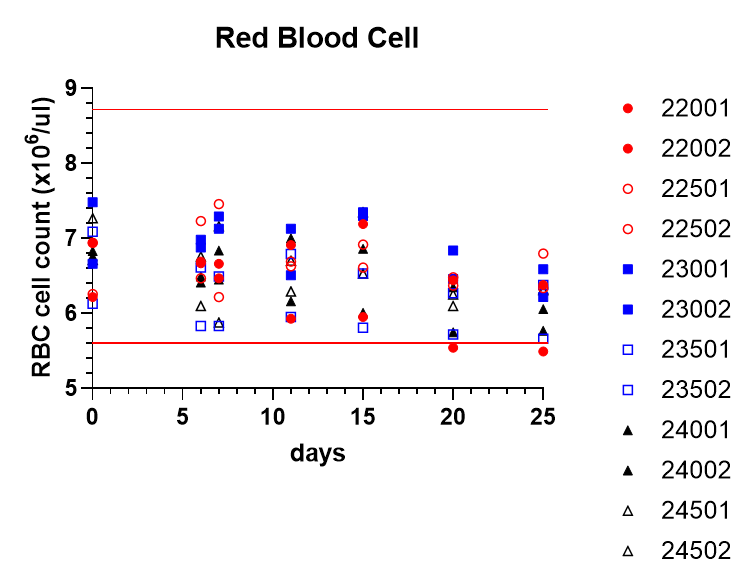

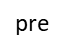

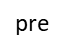

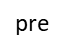

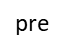

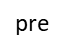

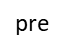


**Supplementary Figure S2.** Selected red blood cell count values of individual peripheral blood

erythrograms for treated dogs over time. Blood samples were obtained 7 days before the

first dose (pre, Y axis) and on study days 6, 7, 11, 15, 20, and following discontinuation of

treatment on day 25 (serial treatment days 4-20). Yellow-shaded area indicates reference

range of values for normal healthy beagle dogs (Charles River Labs). Identification, sex, and

treatment observations for animals given trametinib (red symbols), sapanisertib (blue symbols), and combination treatment (black symbols) are referenced in Figure 5.

**Supplementary Table S1.** Urinalyses performed for dogs receiving trametinib (group 1), sapanisertib (group 2) or the two drugs in combination (group 3) on study days -8 and 25.

Neg, negative; Mod, moderate

d-8, urine specimen collected prior to treatment and d 25, 5 days after conclusion of treatments.


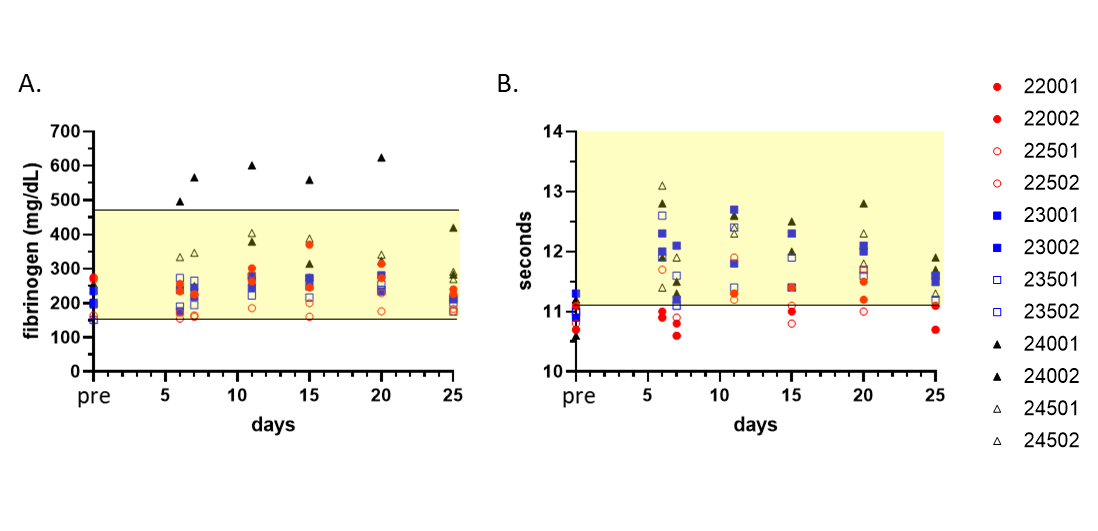


**Supplementary Figure S3.** Fibrinogen **A)** and blood coagulation Activated Partial Thromboplastin Time test (APTT) values **B)** for treated dogs over time. Blood samples were

obtained 7 days before the first dose (pre, Y axis) and on study days 6, 7, 11,

15, 20, and following discontinuation of treatment on day 25 (serial treatment days 4-20). Except for one animal’s fibrinogen levels, all values are within or below yellow-shaded area that indicates reference range of values for normal healthy beagle dogs (Charles River Labs). The upper range of normal APTT values extends above the figure scale

to approximately 19-20 seconds. Identification, sex, and treatment

observations for animals given trametinib (red symbols), sapanisertib (blue

symbols), and combination treatment (black symbols) are referenced in

Figure 5. Treatment group mean APTT values on days 6, 11, 15, 20, 25 for those

dogs receiving sapanisertib alone or in combination were significantly

different from pre-treatment values of all dogs. In addition, APTT from

sapanisertib only and combination-treated dogs were significantly different

from dogs given trametinib alone at 11 days and thereafter (p < 0.05).
